# Clinicopathologic distribution of *KRAS* and *BRAF* mutations in a Chinese population with colorectal cancer precursor lesions

**DOI:** 10.18632/oncotarget.7504

**Published:** 2016-02-19

**Authors:** Chenghao Yi, Yanqing Huang, Xing Yu, Xiaofen Li, Shu Zheng, Kefeng Ding, Jinghong Xu

**Affiliations:** ^1^ Cancer Institute, Key Laboratory of Cancer Prevention and Intervention, China National Ministry of Education, the Second Affiliated Hospital, School of Medicine, Zhejiang University, Hangzhou, China; ^2^ Department of Surgical Oncology, the Second Affiliated Hospital, School of Medicine, Zhejiang University, Hangzhou, China; ^3^ Department of Pathology, the Second Affiliated Hospital, School of Medicine, Zhejiang University, Hangzhou, China

**Keywords:** KRAS, BRAF, serrated lesion, conventional adenoma, colorectal cancer precursor lesions

## Abstract

Investigating the clinical features and corresponding histomorphologic and molecular profiles of precursor lesions of colorectal cancer in a natural population provides new insights into the nature of colorectal cancer, uncovers new screening markers and establishes new prevention strategies for colorectal cancer. In this study, 4302 patients with at least one colorectal polyp from a large colorectal cancer screening program were evaluated and genetic mutations in either *KRAS* or *BRAF* were detected in 495 patients. The population-based mutation rates of *KRAS* and *BRAF* genes in colorectal polyps within this Chinese patient population were 21.8% and 12.1% respectively. Interestingly, considerable variability in the *KRAS* and *BRAF* mutations rates were found among different types of polyps. In a multivariate analysis, presence of villous histology and high-grade dysplasia was associated with *KRAS* mutations (OR, 3.0; 95% CI, 1.7-5.4 and OR, 3.5; 95% CI 1.9-6.5, respectively), while serrated adenomas and hyperplastic polyps were associated with *BRAF V600E* mutations (OR, 20.6; 95% CI, 8.2–51.8 and OR, 11.9; 95% CI 4.9–29.0, respectively). *KRAS* mutations may, in part, drive the histologic progression of adenomas toward a villous histology and higher grades of dysplasia. Mutant *BRAF* may, in part, drive the histologic progression of adenomas toward serrated histology. Dysplasia may arise from hyperplastic polyps, resulting in the formation of serrated adenomas and potentially the development of colorectal carcinoma.

## INTRODUCTION

Colorectal cancer (CRC) is one of the leading causes of cancer death in the world, accounting for over 1.2 million new cancer cases and an estimated 608,700 deaths in 2012[1].

CRC develops through a stepwise evolution from normal mucosa to a precursor lesion and ultimately, a malignant tumor. Adenoma is the primary precursor lesion of CRC [2, 3]. Establishing a risk profile based on histomorphologic features of adenoma is not always straightforward. The addition of molecular profiling provides a more objective and reproducible approach for the classification of colorectal adenomas. The molecular mechanisms underlying the adenoma-carcinoma sequence has been extensively studied and involves a cumulative acquisition of mutations in tumor suppressor genes, such as *APC*, and oncogenes such as *KRAS* and *BRAF,* leading to genomic instability [2, 4-6]. Both *KRAS* and *BRAF* encode kinases that belong to the mitogen-activated protein kinase (*MAPK*) cascade that mediates cellular signaling involved in cell proliferation, apoptosis and differentiation [7]. In adenomas, mutations in *KRAS* and *BRAF* occur in early to advanced adenomas in the adenoma-to-carcinoma sequence. However, the specific role of *KRAS* and *BRAF* mutations in colorectal carcinogenesis remains controversial.

Considering the wide divergence in the frequency of *KRAS* and *BRAF* mutations in the precursor lesions of CRC and the absence of data in the Chinese population, the aims of this study are to investigate the frequency of *KRAS* and *BRAF* mutations in precursor lesions of colorectal cancer in a Chinese population and to study the association between molecular alterations and histologic features.

## RESULTS

### The clinicopathological characteristics of 4302 patients

In this study, the clinicopathologic features of 4302 CRC precursor lesions from a population-based screening was reviewed. In the 4302 patients, 2638 (61.3%) were male, and 1674(38.7%) were female. 748(17.4%) lesions were located in the ascending colon, 762(17.7%) in the transverse colon, 491(11.4%) in the descending colon, 1523(35.4%) in the sigmoid colon, and 778(18.1%) in the rectum. 764(17.8%) lesions were hyperplastic polyps while 3387(78.7%) lesions showed low grade dysplasia, and 151(3.5%) showed high grade dysplasia. Among the 3387 lesions with low grade dysplasia, 2817(65.5%) were tubular adenomas, 462(10.7%) were tubulovillous adenomas, 85(2.0%) were serrated adenomas, and 22(0.5%) were villous adenomas. The characteristics of the colorectal cancer precursor lesions are summarized in Table 1.

**Table 1:** Characteristics of the 4302 colorectal cancer precursor lesions from population-based screening

	Conventional adenomas			Serrated adenomas		HGIN (*n*%)
	TA (*n*%)	TVA (*n*%)	VA (*n*%)	SA (*n*%)	HPs (*n*%)	
Total	2816(65.6)	463(10.8)	22(0.5)	85(2.0)	765(17.8)	151(3.5)
gender						
male	1752(40.7)	299(7.0)	6	55(1.3)	425(9.9)	91(2.1)
female	1064(24.7)	164(3.8)	16(0.4)	30(0.7)	340(7.9)	60(1.4)
location						
Right colon	1096(25.5)	136(3.2)	6	17	217(5.0)	38(0.9)
Left colon	1720(40.0)	327(7.6)	16	68(1.9)	548(12.7)	113(2.6)
age						
<40	31(0.7)	3	0	0	10	0
40-49	490(11.4)	69(1.6)	1	13	152(3.5)	16
50-59	976(22.7)	150(3.5)	8	29	266(6.2)	50(1.2)
60-69	995(23.1)	171(4.0)	12	34	259(6.2)	66(1.5)
70-79	308(7.2)	66(1.5)	1	8	74(1.7)	16
≥80	16(0.4)	4	0	1	4	3

TA= Tubular adenoma, TVA= Tubulovillous adenoma, SA= Serrated adenoma, VA= Villous adenoma, HPs= Hyperplastic polyps, HGIN= High grade intraepithelial neoplasia

### The clinicopathologic features of the selected patients

A total of 495 subjects with at least one colorectal cancer precursor lesion were selected for genotyping of *KRAS* and *BRAF*. Of the 495 subjects, 311 were male, and 184 were female. The patients' ages ranged from 20 to 88 years with the mean age of 60 years. There were 346(69.9%) lesions located in the left colon, 149(30.1%) lesions located in the right colon, 65(13.1%) hyperplastic polyps, 337(68.1%) low grade intraepithelial neoplasia, and 93(18.8%) high grade intraepithelial neoplasia. In 337 low grade intraepithelial neoplasia, 155(31.3%) were tubular adenomas, 123(24.8%) were tubulovillous adenomas, 52(10.5%) were serrated adenomas (44 were TSAs, and 8 were SSA/Ps), and 7(1.4%) were villous adenomas.

### Distributions of KRAS mutations in the selected patients

Among the 495 colorectal polyps and adenomas, 143 were identified as carrying *KRAS* codon12 or 13 mutations, which was 28.9% of the total polyps and adenomas surveyed. In the 143 subjects with mutant *KRA*S, 93 (93/311, 29.9%) were male, and 50 (50/184, 27.2%) were female. 46(46/149, 30.9%) were located in the right colon, and 97(97/346, 28.0%) were located in the left colon. 20(20/65,30.8%) were hyperplastic polyps, 85(85/337, 25.2%) were low grade intraepithelial neoplasia, and 38(38/93, 40.8%) were high grade intraepithelial neoplasia. In the low grade intraepithelial neoplasias with mutant *KRAS*, 46(46/123,37.4%)were tubulovillous adenomas, 24(24/155,15.5%) were tubular adenomas, 12(12/52,23.1%)were serrated adenomas, and 3(3/7,42.9%) were villous adenomas (Table 2).

**Table 2:** Distributions of *KRAS* mutations in the selected patients

Clinical characteristic	*n*	*KRAS* mutation (n%)	Univariate OR (95%CI)	Multivariate OR (95%CI)
Total	495	143(28.9%)		
gender				
women	184	50(27.1%)	1(reference)	1(reference)
men	311	93(29.9%)	1.1(0.8-1.7)	1.2(0.8-1.8)
age				
≤53	137	27(19.7%)	1(reference)	1(reference)
54-60	124	36(29.0%)	1.7(1.0-3.0)	1.6(0.9-3.0)
61-66	119	46(38.6%)	2.6(1.5-4.5)	2.3(1.3-4.1)
>66	115	33(28.6%)	1.6(0.9-2.9)	1.5(0.8-2.7)
location				
Right colon	149	46(30.9%)	1(reference)	1(reference)
Left colon	346	97(28.0%)	0.9(0.6-1.3)	0.9(0.5-1.4)
histology				
TA	155	24(15.5%)	1(reference)	1(reference)
TVA	123	46(37.4%)	3.3(1.8-5.8)	3.0(1.7-5.4)
SA	52	12(23.1%)	1.6(0.8-3.6)	1.5(0.7-3.2)
VA	7	3(43.0%)	4.1(0.9-19.4)	3.8(0.8-18.4)
HPs	65	20(30.8%)	2.4(1.2-4.8)	2.3(1.2-4.6)
HGIN	93	38(40.9%)	3.8(2.1-6.9)	3.5(1.9-6.5)

TA= Tubular adenoma, TVA= Tubulovillous adenoma, SA= Serrated adenoma, VA= Villous adenoma, HPs= Hyperplastic polyps, HGIN= High grade intraepithelial neoplasia

### Distribution of different KRAS mutations

Of the 143 adenomas with *KRAS* mutations, 101 (70.6%) had a single mutation at codon 12; 30 (21.0%) had a single mutation at codon 13; and 12 (8.4%) had 2 different *KRAS* mutations. Of the 155 *KRAS* mutations identified, 97 (62.6%) were transitions and 58 (37.4%) were transversions. The most common single mutation was a GGT to a GAT transition in codon 12 that resulted in a change from the amino acid glycine to aspartic acid. This particular mutation occurred in 43.4% of the colorectal cancer precursor lesions with KRAS mutations (Table 3).

**Table 3:** Distributions of different type mutations of *KRAS*

Codon12(AA)	Codon13(AA)	Mutation type	*n*%
Single mutations			
GAT(Asp)		transition	62(43.4%)
GTT(Val)		transversion	27(18.9%
TGT(Cys)		transversion	8(5.6%)
GCT (Ala)		transversion	4(2.8%)
	GAC (Asp)	transition	28(19.6%)
	TGC (Cys)	transversion	1(0.7%)
	AGC (13Ser)	transversion	1(0.7%)
Double mutations			
GAT(Asp)	GAC (Asp)	Transition	6(4.2%)
GAT;GTT(Asp; Val)		Transition/transversion	3(2.1%)
AGT ;GCT(Ser;Ala)		transversion	1(0.7%)
GAT;GCT(Asp; Ala)		Transition/transversion	1(0.7%)
GAT(Asp)	TGC (Cys)	Transition/transversion	1(0.7%)

AA=amino acids

### Univariate OR and Multivariate OR for KRAS mutations

There were no differences in the prevalence of *KRAS* mutations by gender as well as by location of the colorectal cancer precursor lesions (Table 2). Older participants were more likely to have a precursor lesion with a *KRAS* mutation (24.5% in < 60 *vs* 33.1% in ≥60, *P* = 0,04). A strong relationship was found between *KRAS* mutations and precursor lesion histology. *KRAS* mutations were present in 38.8% of the adenomas that were tubulovillous or villous compared with 15.5% of adenomas that were of tubular histology. Tubulovillous and villous adenomas were combined for this analysis because there were only 7 purely villous adenomas in the study for separate analyses, but the frequency of mutations in the 7 villous adenomas was 42.9%. *KRAS* mutations presented in 40.9% of adenomas that were graded as having high-grade dysplasia, but only in 25.2% of adenomas with low-grade dysplasia.

In the multivariate analyses (Table 2), presence of villous histology and high-grade dysplasia remained significantly and independently associated with *KRAS* mutations (OR, 3.0; 95% CI, 1.7-5.4 and OR, 3.5; 95% CI 1.9-6.5, respectively).

### Distribution of BRAF mutations in the selected patients

A total of 77 colorectal cancer precursor lesions were identified with *BRAF* V600E mutations, 45 from male subjects, and 32 from female subjects. 18(18/149,12.1%) were located in the right colon, 59(59/346,17.1%)were located in the left colon. 24(24/65, 36.9%) were hyperplastic polyps, 48 were low grade intraepithelial neoplasia, and 5 were high grade intraepithelial neoplasia. In low grade intraepithelial neoplasia with mutant *BRAF*, 10 were tubulovillous adenomas, 8 were tubular adenomas, 26(26/52,50.0%) were serrated adenomas, and 4(4/7,57.1%) were villous adenomas.

Of the 77 *BRAF* V600E mutations, 64.9% (50/77) present in serrated lesions (50.0% in serrated adenomas and 36.9% in hyperplastic polyps). There were only 7.1% (27/378) *BRAF* V600E mutations present in other types of colorectal adenomas (tubular, tubulovillous and villous adenomas). We found only 5 *BRAF* V600E mutations present in high grade intraepithelial neoplasia.

### Univariate OR and multivariate OR for BRAF mutations

There were no differences in the prevalence of *BRAF* mutations by gender, age or location of precursor lesions. In the multivariate analyses (Table 4), the presence of serrated histology and hyperplastic polyps remained significantly and independently associated with *BRAF* V600E mutations (OR, 20.6; 95% CI, 8.2-51.8 and OR, 11.9; 95% CI 4.9-29.0, respectively).

We found 7 patients with both *KRAS* codons 12 and 13 mutations as well as *BRAF* V600E mutations within the same precursor lesion.

**Table 4:** Distributions of *BRAF* mutations in the selected patients

Clinical characteristic	*n*	*BRAF* mutation (*n*%)	Univariate OR (95%CI)	Multivariate OR (95%CI)
Total	495	143(28.9%)		
sex				
women	184	32(17.4%)	1(reference)	1(reference)
men	311	45(14.4%)	0.8(0.5-1.3)	0.7(0.4-1.2)
age				
≤53	137	15(10.9%)	1(reference)	1(reference)
54-60	124	24(19.4%)	1.4(0.7-2.8)	1.8(0.8-4.0)
61-66	119	21(17.6%)	1.4(0.7-2.8)	1.3(0.6-3.0)
>66	115	17(14.8%)	1.1(0.5-2.1)	1.0(0.4-2.2)
location				
Right colon	149	18(12.1%)	1(reference)	1(reference)
Left colon	346	59(17.1%)	1.5(0.85-2.6)	1.6(0.9-2.7)
histology				
TA	155	8(5.2%)	1(reference)	1(reference)
TVA	123	10(8.1%)	1.5(0.6-4.3)	1.8(0.7-4.7)
SA	52	26(50.0%)	18.4(7.5-45)	20.6(8.2-51.8)
VA	7	4(57.0%)	24.5(4.7-128.5)	26.2(4.7-144.2)
HPs	65	24(36.9%)	10.8(4.5-25.7)	11.9(4.9-29.0)
HGIN	93	5(5.3%)	1.0(0.3-3.3)	1.1(0.3-3.5)

TA= Tubular adenoma, TVA= Tubulovillous adenoma, SA= Serrated adenoma, VA= Villous adenoma, HPs= Hyperplastic polyps, HGIN= High grade intraepithelial neoplasia

## DISCUSSIONS

Several risk factors for colorectal cancer have been identified by epidemiologic studies, including characteristics of both the host (age, gender, race, and family cancer history) [8, 9] and the environment (consumption of red and processed meats, smoking, alcohol consumption, diabetes mellitus, low level of physical activity and obesity) [10-17]. Similarly, several characteristics of colorectal cancer precursor lesions (size, location, histology, and degree of dysplasia) have been found to be associated with an increased risk of precursor progression to carcinoma [18, 19]. In parallel, numerous studies have revealed that cancer results, in part, from the accumulation of genetic alterations, including activating mutations of proto-oncogenes, inactivating mutations of tumor suppressor and DNA repair genes, DNA-methylation and chromatin structure changes [20-22]. Relatively little is known about the relationship amongst host and environmental risk factors, colorectal cancer precursor lesions characteristics, and genetic events, and how they work together to drive the process of carcinogenesis.

*KRAS* and *BRAF* mutations occur relatively early in the adenoma-carcinoma process [23-26]. Somatic mutations that activate regulators and effectors of RAS proteins are common in tumor development [1-3]. In approximately 35.0%-42.0% of early colorectal cancer (CRC) pa­tients, *KRAS* mutations inhibit *KRAS* GTPase, resulting in constitutive activation of *KRAS,* and in turn, activation of the *RAS/RAF* signaling pathway. In CRC, 97.0% of *KRAS* mutations occur in codons 12 and 13 of exon 2 [4]. *BRAF* is a human gene that encodes the oncoprotein *BRAF*, a serine/threonine protein kinase [5]. *BRAF* is a member of the *RAF* kinase family that regulates the *RAS/RAF/MEK/*extracellular signal regulated kinase (ERK) pathway and is involved in cell division, differentiation, and secretion [6]. The most common *BRAF* mutation is a missense mutation (V600E), resulting in the replacement of valine for glutamic acid which generates aberrant MEK/ERK signaling in CRC [7]. Numerous large studies have investigated the clinical significance of *KRAS and BRAF* mutations in CRC [4-6, 27-31], but the specific role of *KRAS* and *BRAF* mutations in colorectal carcinogenesis remains unclear. In this study, we investigated several aspects of the molecular epidemiology of *KRAS and BRAF* mutations in sporadic colorectal cancer precursor lesions.

Amongst the 4302 patients examined with at least one colorectal cancer precursor lesion, the male to female ratio was approximately1.6:1. Studies have indicated male hormones increase the incidence of colonic polyps [32, 33]. The differences of lifestyle between genders including smoking and alcohol consumption may also contribute to this increased incidence in males [13, 19]. Amongst the adenomas examined, 491(11.4%) were located in the descending colon, 1523(35.4%) in the sigmoid colon, and 778(18.1%) in the rectum. This data reveals that the majority of precursor lesions are located in the left colon (64.9%) as similarly reported in previous studies [18, 34, 35]. Histopathologic subtyping of these precursor lesions revealed that tubular and tubulovillous adenomas accounted for 76.2% (3279/4302), while hyperplastic polyps and serrated adenomas accounted for 19.3% (849/4302). There were 8 SSA/Ps amongst the 495 patients. Previous studies from a Western population showed that SSAs constitute approximately 9.0% of all polyps and 22.0% of serrated polyps [36, 37]. Thus, the proportion of SSAs in the present study is much lower in comparison to that in Western patients. A lower proportion of SSAs in the Chinese population has been reported by Qiu et al (SSAs accounted for 4.9% of serrated polyps and 1.0% of all colorectal polyps according to their data) [38]. This great variation in SSA/P detection rates between this and previous studies can be caused by genetic factors, lifestyle and dietary factors amongst the different patient populations.

We found that 28.9% of the 495 colorectal cancer precursor lesions had mutations in the *KRAS* gene which was in range with the 15.0%-75.0% prevalence of *KRAS* mutations in polyps reported in previous studies [39-51]. It is likely that this wide range reflects differences in race and patient selection. The *KRAS* mutation rate in our study was approximately 21.8% by standardized rate which is similar to the result of Nusko et al[46].

We found that individuals over the age of 60 years had about a 1.3-fold increase in risk of having a *KRAS* mutant colorectal cancer precursor lesion than younger individuals in a univariate analysis. However the multivariate analysis showed that age was not an independent predictor for *KRAS* mutation in colorectal cancer precursor lesions. The absolute frequency of *KRAS* mutations in right colon lesions is higher than that in the left (30.9% *vs* 28.0%), but this difference did not reach statistical significance (*P* = 0.52).

In our univariate analysis, villous histology and high grade dysplasia are each significantly associated with *KRAS* mutations. The result is similar to Maltzman T et al [50]. A strong association between *KRAS* mutations and both villous histology and high grade dysplasia persisted after multivariate analysis of our data. These observations suggest the possibility that *KRAS* mutations may, in part, drive the histologic progression of adenomas toward a villous histology and higher grades of dysplasia.

In our data, *BRAF* mutations were detected in 50.0% of serrated adenomas, 36.9% of hyperplastic polyps and 7.1% of other types of colorectal cancer precursor lesions. The *BRAF* mutation rates in serrated adenomas and hyperplastic polyps were lower than that reported in previous studies [37, 45, 51-55]. The overall *BRAF* mutation rate in colorectal cancer precursor lesions in this studied population was 12.1% by standardized rate, similar to previous studies [56, 57]. A strong association was found between *BRAF* mutations and both serrated adenomas and hyperplastic polyps during multivariate analysis. The similar *BRAF* mutation rates between serrated adenomas and hyperplastic polyps suggests that dysplasia may arise from hyperplastic polyps, resulting in the formation of serrated adenomas and potentially the development of colorectal carcinoma.

The traditional view of hyperplastic polyps (HPs) is that they are relatively common, non-neoplastic lesions that may be safely ignored and are distinctly separate from the smaller category of neoplastic polyps and precancerous adenomas [58]. There were 65 hyperplastic polyps in our study with 39(60.0%) located in the sigmoid colon and rectum. We found 20 *KRAS* mutations and 24 *BRAF* mutations in these 65 hyperplastic polyps. On histopathologic examination, hyperplastic polyps show a serrated morphology [59, 60]. In cancer progression, molecular changes tend to precede morphologic changes. Although dysplasia is not seen in hyperplastic polyps, it would be interesting to further evaluate the relationship between HPs and serrated adenoma in future studies.

The characteristic distribution of *KRAS* and *BRAF* mutations suggest there may be two different pathways in adenoma to adenocarcinoma progression. The first pathway is a traditional adenoma-carcinoma sequence involving *KRAS* mutations. Mutant *KRAS* may, in part, drive the histologic progression of adenomas toward villous histology and higher grades of dysplasia. Another proposed pathway is a serrated adenoma- carcinoma sequence. The polyps of this pathway would differ morphologically and genetically from those of a traditional adenoma-carcinoma sequence. Mutant *BRAF* may, in part, drive the histologic progression of adenomas toward serrated histology. In turn, the proposed two different adenoma-carcinoma sequences would then likely result in potentially different colorectal carcinoma subtypes. In the 2012 Cancer Genome Atlas Network study [61], *KRAS* mutations presented in 43.0% of non-hypermutated colorectal cancers and 30.0% of hypermutated colorectal cancers while, *BRAF* mutations presented in 3.0% of non-hypermutated colorectal cancers and 47.0% of hypermutated colorectal cancers. We found striking similarities between colorectal cancers and adenomas during the evaluation of *KRAS* and *BRAF* mutations (Table 5). Therefore, we propose a traditional adenoma-carcinoma sequence results in non-hypermutated colorectal cancers while a serrated adenoma-carcinoma sequence results in hypermutated colorectal cancers. Further investigation is needed to validate this hypothesis and support the preliminary data presented in this study.

Currently, the treatment decisions of colorectal cancer is based on the TNM classification system which ignores molecular findings. The prognosis of colorectal cancer patients may be improved by taking into consideration molecular pathological features and biological characteristics of different colorectal cancer subtypes.

**Table 5:** Distributions characteristic of *KRAS* and *BRAF*

	Total	Conventional adenomas			Serrated lesions			HGIN
		TA	TVA	VA	TSA	SSA/P	HPs	
Total	495	155	123	7	44	8	65	93
KRAS mutations	143(28.9%)	24(15.5%)	46(37.4%)	3	11(25.0%)	1	20(30.8%	38(40.9%)
BRAF mutations	77(15.5%)	8(5.2%)	10(8.1%)	4	20(45.5%)	6	24(36.9%)	5(5.4%)

TA= Tubular adenoma, TVA= Tubulovillous adenoma, VA= Villous adenoma, TSA= Traditional serrated adenoma, SSA/P= Sessile serrated adenoma/polyp, HPs= Hyperplastic polyps, HGIN= High grade intraepithelial neoplasia

*KRAS* mutation present in 25.6% traditional adenomas and 27.4% serrated adenomas, *BRAF* mutation present in 7.1% traditional adenomas and 42.7% serrated adenomas. In the multivariate analyses, presence of villous histology and high-grade dysplasia independently associated with *KRAS* mutations (OR, 3.0; 95% CI, 1.7–5.0 and OR, 3.5; 95% CI 1.5–6.4, respectively), presence of serrated histology and hyperplastic polyps independently associated with *BRAF V600E* mutations (OR, 20.6; 95% CI, 8.2–51.8 and OR, 11.9; 95% CI 4.9–29.0, respectively).

## SUMMARY

The process of carcinogenesis is thought to be driven, in part, by sequential rounds of mutations followed by clonal expansion. The final histologic and biologic features of adenomas are the result of the accumulation of a series of molecular events. Knowledge of the mutational profile of a precancerous lesion may be expected to provide greater insight into its clinical behavior in comparison to histology or size of the lesion. The present study has several clinical implications. It provides a molecular basis for the clinical observations that hyperplastic polyps may have malignant potential. Furthermore, *KRAS* and *BRAF* are likely involved in the malignant transformation of colorectal adenomas as driver genes. In the future, *KRAS* and *BRAF* may serve as molecular markers for colorectal screening and molecular typing as well as potential targets for therapeutic intervention.

## MATERIALS AND METHODS

### Patients

The patients were recruited from a population-based colorectal cancer screening program. A total of 87254 subjects between 40 to 74 years old were screened by questionnaire-based risk assessment and fecal immunochemical testing from May 2011 to April 2014. Among them, a total of 4302 subjects with at least one CRC precursor lesions were accepted for endoscopic resection. Hematoxylin and Eosin (H&E)-stained slides from formalin-fixed paraffin-embedded (FFPE) lesion samples (*n* = 4302) were reviewed by pathologists. In the 4302 lesions, 495 lesions were selected and analyzed for *KRAS* and *BRAF* genotyping. The right colon was from ileocecal valve to splenic flexure of the colon. The left colon was from the splenic flexure of colon to rectum. This study was approved by the Committee for the Protection of Human Subjects at the Second Affiliated Hospital, School of Medicine, Zhejiang University.

### DNA extraction

Genomic DNA from 495 precursor lesions of CRC samples was extracted from freshly cut formalin-fixed, paraffin-embedded tissue sections (five 10-μm sections) using a QIAamp DNA FFPE Tissue Kit (Qiagen, Hilden, Germany) according to the manufacturer's instructions. The lesion area was identified through haematoxylin-eosin staining, and tissue from this area on unstained sections was removed for DNA extraction into microcentrifuge tubes.

### Analysis of KRAS and BRAF mutations

To detect genetic alterations in *KRAS* and *BRAF*, we analyzed the point mutations of codons 12 and 13 in the *KRAS* gene and the mutation of exon 15 codon 600 in the *BRAF* gene by direct sequencing. The primers for *KRAS* sequencing of codons 12 and 13 were as follows: 5′-GGTACTGGAGTATTTGAT-3′ (forward) and 5′-TTGTGGACGAATATGATCCAACA-3′ (reverse). The primers for sequencing the *BRAF* exon 15 codon 600 were as follows: 5′-TCATAATGCTTGCTCTGATAGGA-3′ (forward) and 5′-TCCACTGATTAAATTTTTGGCC-3′ (reverse).

Amplification of codons 12 and 13 of *KRAS* gene and the exon 15 codon 600 of *BRAF* gene was carried out in a Touchgene Gradient Thermal Cycler (Techne Inc., Princeton, NJ) in a 25 μl PCR reaction mixture containing 1μl genomic DNA, 1 μl forward primers, 1 μl reverse primers, 12.5 μl KAPA2G (KAPA2G, Wilmington, MA) Fast Multiplex Mix and 9.5 μl ddH_2_O.

The PCR reaction for codons 12 and 13 of the *KRAS* gene was as follows: 94°C, 5 minutes; and 30 cycles of 94°C, 30 seconds; 56°C, 30 seconds; 72°C, 1 minute; and 72°C, 5 minutes. The 201 base pair PCR product was then visualized on a 2.0% agarose gel. DNA samples that failed to amplify with 1 μl were repeated using 5 μl of the extracted DNA. The PCR reaction for the exon15 codon 600 of *BRAF* gene was as follows: 94°C, 5 minutes; and 30 cycles of 94°C, 30 seconds; 55°C, 30 seconds; 72°C, 1 minute; and 72°C, 5 minutes. The 215 base pair PCR product was then visualized on a 2.0% agarose gel. DNA samples that failed to amplify with 1 μl were repeated using 5 μl of the extracted DNA. For each set of samples extracted, negative control tubes containing no tissue were simultaneously extracted and tested for ability to amplify a product. Known positive and negative control samples were regularly analyzed.

Direct sequencing was performed using an ABI 3730XL DNA Analyzer (Applied Biosystems, Foster City, California, USA). PCR and sequence analysis of mutated samples were repeated twice to exclude PCR errors.

### Statistical analysis

The relationship between *KRAS* codon 12 or 13 mutations and *BRAF* V600E in colorectal cancer precursor lesions and various characteristics of patients and corresponding lesions were assessed by both univariate and multiple logistical analysis using IBM SPSS software for Windows, version 19.0 (IBM Corporation, Armonk, New York). Odds ratios (ORs) were first calculated separately for all of the independent variables (patient and adenoma characteristics) with *KRAS* and *BRAF* mutational status (wild-type *vs*. mutant) as the independent variable. Multiple logistic models that included all lesions or all patient characteristics were then performed for both specimens-based and patients-based analysis. Multiple logistic regression models of specimens-based analysis were estimated using generalized estimating equations to allow potential correlation among the adenomas within the same individual. Standardized rate was used to estimate the mutation rate of *KRAS* and *BRAF* in population. The number of decimal places in percentages was set to one.
